# Trimmomatic: a flexible trimmer for Illumina sequence data

**DOI:** 10.1093/bioinformatics/btu170

**Published:** 2014-04-01

**Authors:** Anthony M. Bolger, Marc Lohse, Bjoern Usadel

**Affiliations:** ^1^Department Metabolic Networks, Max Planck Institute of Molecular Plant Physiology, Am Mühlenberg 1, 14476 Golm,^2^Institut für Biologie I, RWTH Aachen, Worringer Weg 3, 52074 Aachen and ^3^Institute of Bio- and Geosciences: Plant Sciences, Forschungszentrum Jülich, Leo-Brandt-Straße, 52425 Jülich, Germany

## Abstract

**Motivation:** Although many next-generation sequencing (NGS) read preprocessing tools already existed, we could not find any tool or combination of tools that met our requirements in terms of flexibility, correct handling of paired-end data and high performance. We have developed Trimmomatic as a more flexible and efficient preprocessing tool, which could correctly handle paired-end data.

**Results:** The value of NGS read preprocessing is demonstrated for both reference-based and reference-free tasks. Trimmomatic is shown to produce output that is at least competitive with, and in many cases superior to, that produced by other tools, in all scenarios tested.

**Availability and implementation:** Trimmomatic is licensed under GPL V3. It is cross-platform (Java 1.5+ required) and available at http://www.usadellab.org/cms/index.php?page=trimmomatic

**Contact:** usadel@bio1.rwth-aachen.de

**Supplementary information:** Supplementary data are available at *Bioinformatics* online.

## 1 INTRODUCTION

The presence of poor quality or technical sequences such as adapters in next-generation sequencing (NGS) data can easily result in suboptimal downstream analyses.

Nonetheless, it is not trivial to precisely identify such sequences, including partial adapter sequences, while leaving valid sequence data intact (Li *et al.*, 2013). Furthermore, given the rate with which NGS sequence data are currently being produced (Mardis, 2008), the additional burden of sequence preprocessing must be kept relatively modest so as to avoid adding undue overhead to the bioinformatics pipeline.

The preprocessing approach must also not interfere with the downstream analysis of the data. For example, NGS data often come in the form of paired-end reads, and typically, the forward and reverse reads are stored in two separate FASTQ files, which contain reads from each DNA fragment in the same order. Many downstream tools use this positional relationship between pairs, so it must be maintained when preprocessing the sequence data.

The wide range of available NGS library preparations combined with the range of downstream applications demand a flexible approach. It should be possible to choose a set of processing steps to be applied in a user-defined order, and ideally even allow some steps to be included more than once. In other domains, this can be achieved using a shell pipeline to combine multiple tools as required, e.g. in Newick (Junier and Zdobnov, 2010). However, the need for ‘pair awareness’ makes this approach difficult to apply, as the connection between the corresponding reads in the paired files will typically be lost. Correcting this would require an additional step to reconcile the read pairs and store the ‘singleton’ reads separately. Furthermore, the processing steps would not be able to assess the read pair as a unit, which is necessary or at least advantageous in some cases.

The alternative approach of executing a series of tools in succession would involve the creation of intermediate files at each step, a non-trivial overhead given the data size involved, and would still require pair-awareness to be built into every tool used. These issues suggest that the typical approaches to achieve flexibility by combining multiple single-purpose tools are not optimal.

Thus, although many NGS read preprocessing tools exist, none of them, alone or in combination, could offer the desired flexibility and performance, and most were not designed to work on paired-end data. As a result, we developed Trimmomatic as a more flexible, pair-aware and efficient preprocessing tool, optimized for Illumina NGS data.

## 2 ALGORITHMS

Trimmomatic includes a variety of processing steps for read trimming and filtering, but the main algorithmic innovations are related to identification of adapter sequences and quality filtering, and are described in detail below. A list of the other processing steps is presented in the Supplementary Materials.

### 2.1 Removal of technical sequences

Trimmomatic uses two approaches to detect technical sequences within the reads. The first, referred to as ‘simple mode’, works by finding an approximate match between the read and the user-supplied technical sequence. This mode has the advantage of working for all technical sequences, including adapters and polymerase chain reaction (PCR) primers, or fragments thereof. Such sequences can be detected in any location or orientation within the reads but requires a substantial minimum overlap between the read and technical sequence to prevent false-positive findings. However, short partial adapter sequences, which often occur at the ends of reads, are inherently unable to meet this minimum overlap requirement and therefore are not detectable.

The second mode, referred to as ‘palindrome mode’, is specifically aimed at detecting this common ‘adapter read-through’ scenario, whereby the sequenced DNA fragment is shorter than the read length, and results in adapter contamination on the end of the reads. This is especially the case for longer read length as supported by the Miseq. Although such short fragments should normally be removed during library preparation, in practice this process is not perfectly efficient, and thus many libraries suffer from this problem to some extent. ‘Palindrome mode’ can only be used with paired-end data, but has considerable advantages in sensitivity and specificity over ‘simple’ mode.

Note that the current technical sequence identification approaches in Trimmomatic are not designed to filter or categorize data on the basis of ‘barcodes’.

#### 2.1.1 Simple mode

In simple mode, each read is scanned from the 5′ end to the 3′ end to determine if any of the user-provided adapters are present. The standard ‘seed and extend’ approach (Li and Homer, 2010) is used to find initial matches between the technical sequences and the reads. The seed is not required to match perfectly, and a user-defined number of mismatches are tolerated. Based on this seed match, a local alignment is performed. If the alignment score exceeds the user-defined threshold, the aligned region plus the remainder after the alignment are removed.

**Fig. 1.**
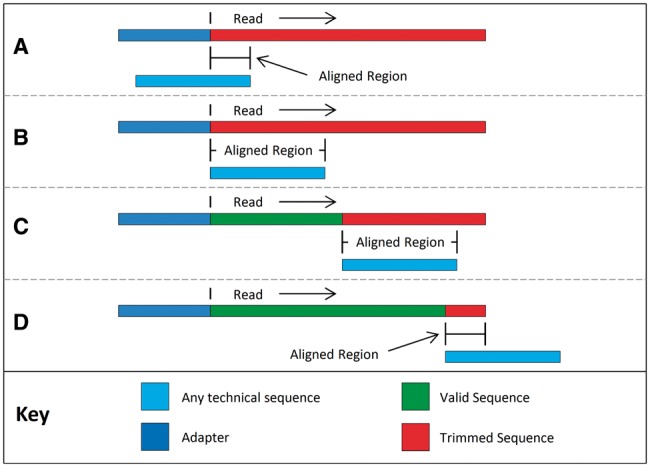
Putative sequence alignments as tested in simple mode. The alignment process begins with a partial overlap at the 5′ end of the read (**A**), increasing to a full-length 5′ overlap (**B**), followed by full overlaps at all positions (**C**) and finishes with a partial overlap at the 3′ end of the read (**D**).**** Note that the upstream ‘adapter’ sequence is for illustration only and is not part of the read or the aligned region****

Figure 1 illustrates the alignments tested for each technical sequence. The process begins with a partial overlap of the 3′ end of the technical sequence with the 5′ end of the read, as shown in (A). Testing proceeds by moving the putative contaminant toward the 3′ end of the read. In both the partial overlap (A) and complete overlap at the 5′ end (B) scenarios, the entire read will be clipped. If the contaminant is found within the read (C), the bases from the 5′ end of the read to the beginning of the alignment are retained. The testing process continues until only a partial alignment on the 3′ end of the read remains (D).

Simple mode has the advantage that it can detect any technical sequence at any location in the read, provided that the alignment is sufficiently long and the read is sufficiently accurate. However, when only a short partial match is possible, such as in scenarios (A) and (D), the contaminant may not be reliably detectable.

#### 2.1.2 Palindrome mode

As noted above, ‘palindrome mode’ is specifically optimized for the detection of ‘adapter read-through’. When ‘read-through’ occurs, both reads in a pair will consist of an equal number of valid bases, followed by contaminating sequence from the ‘opposite’ adapters. Furthermore, the valid sequence within the two reads will be reverse complements. By detecting all three of these symptoms at once, adapter read-through can be identified with high sensitivity and specificity.

For performance reasons, the actual algorithm combines these three tests. The adapter sequences are prepended to their respective reads, and then the combined read-with-adapter sequences from the pair are aligned against each other. A high-scoring alignment indicates that the first parts of each read are reverse complements, while the remaining parts of the reads match the respective adapters.

The alignment is implemented using a ‘seed and extend’ approach, similar to that in simple mode. Global alignment scoring is used to ensure an end-to-end match across the entire overlap.

**Fig. 2.**
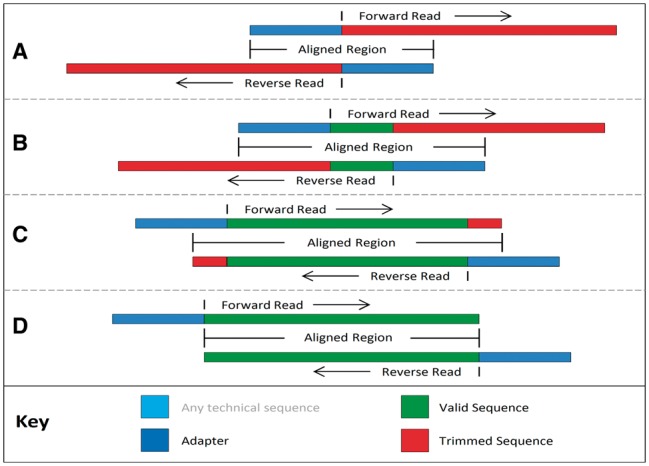
Putative sequence alignments as tested in palindrome mode. The alignment process begins with the adapters completely overlapping the reads (**A**) testing for immediate ‘read-through’, then proceeds by checking for later overlap (**B**), including partial adapter read-through (**C**), finishing when the overlap indicates no read-through into the adapters (**D**)

Figure 2 illustrates the alignments tested in palindrome mode. The process begins with an overlap between the adapters and the start of the opposite reads, as shown in (A). This alignment would detect a read pair containing no useful sequence information, which could be caused by the direct ligation of the adapters. Detection of this scenario would result in the dropping of both reads. Testing then proceeds by moving the relative positioning of the reads ‘backwards’, testing for increasingly longer valid DNA fragments, illustrated in (B). This scenario would result in the trimming of both reads as illustrated. Even when only a small fragment of the adapter is overlapping, as shown in (C), the overall alignment is easily sufficient to ensure reliable detection. The process is complete when the overlapping region no longer reaches into the adapters (D).

The main advantage of palindrome mode is the longer alignment length, which ensures that the adapters can be reliably detected, even in the presence of read errors or where only a small number of adapter bases are present. If required, palindrome mode can be used to remove even a single adapter base, while retaining a low false-positive rate. We are aware of one other tool, AdapterRemoval (Lindgreen, 2012), which independently developed a similar approach.

Note, however, because palindrome is limited to the detection of adapter read-through, a comprehensive strategy requires the combination of both simple and palindrome modes.

#### 2.1.3 Alignment detection and scoring

The algorithmic approach used for technical sequence alignments is somewhat unusual, avoiding the precalculated indexes often used in NGS alignments (Li and Homer, 2010).

Initial sequence comparisons are done using a 16-base fragment from each sequence. The 16 bases are converted to the 64-bit integer, known as the seed, using a 4-bit code for each base: A = 0001, T = 0010, C = 0100 and T = 1000. These seeds are then compared using a bitwise-XOR, which determines which bits differ between the two seeds. This will result in a 0000 code for each matching base, and a code with two 1 s for each mismatch, e.g. 0011 for an A-T mismatch, as XOR(0001,0010) = 0011. The ‘1’s within this result are then counted using the ‘popcount’ operation, and this count will be exactly twice the number of differing bases for the 16-base fragments.

If the seeds are within the user-specified distance, the full alignment scoring algorithm is used. Matching bases are scored as 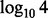, which is ∼0.602, while mismatches are penalized depending on their quality score, by 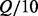, which can thus vary from 0 to 4. This results in a higher penalty for bases that are believed to be highly accurate.

‘Simple’ mode aligns each read against each technical sequence, using local alignment. This is implemented by finding the highest scoring region within the alignment, and thus may omit divergent regions on the ends.

‘Palindrome’ mode aligns the forward and reverse reads, combined with their adapter sequences. It uses global alignment, which is the total alignment score of the overlapping region.

### 2.2 Quality filtering

Trimmomatic offers two main quality filtering alternatives. Both approaches exploit the Illumina quality score of each base position to determine where the read should be cut, resulting in the retention of the 5′ portion, while the sequence on the 3′ of the cut point is discarded. This fits well with typical Illumina data, which generally have poorer quality toward the 3′ end. These two approaches are described in the following sections.

#### 2.2.1 Sliding Window quality filtering

The Sliding Window uses a relatively standard approach. This works by scanning from the 5′ end of the read, and removes the 3′ end of the read when the average quality of a group of bases drops below a specified threshold. This prevents a single weak base causing the removal of subsequent high-quality data, while still ensuring that a consecutive series of poor-quality bases will trigger trimming.

#### 2.2.2 Maximum Information quality filtering

A novel alternative approach was motivated by the realization that, for many applications, the incremental value of retaining additional bases in a read is related to the read length. Intuitively, it is clear that short reads are almost worthless because they occur multiple times within the target sequence and thus they give only ambiguous information. Even at the risk of introducing errors, it is worthwhile to retain additional low-quality bases early in a read, so that the trimmed read is sufficiently long to be informative.

However, beyond a certain read length, retaining additional bases is less beneficial, and may even be detrimental. Reads of moderate length are likely to be already informative and, depending on the task at hand, can be almost as valuable as full-length reads. Therefore, the smaller potential benefit of retaining additional bases must be balanced against the increasing risk of retaining errors, which could cause the existing read value to be lost.

As such, it is worthwhile for the trimming process to become increasingly strict as it progresses through the read, rather than to apply a fixed quality threshold. To the best of our knowledge, this approach has not been applied in any existing tools.

The ‘Maximum Information’ quality filtering approach implements this adaptive approach. It uses a combination of three factors to determine how much of each read should be retained.

The first factor models the ‘length threshold’ concept, whereby a read must be of at least a minimal length to be useful for the downstream application. As described above, very short reads have little value, as they are too ambiguous to be informative. On the other hand, most long reads can be mapped to few locations in the target sequence. If they cannot be uniquely mapped, because of them originating in a repetitive region, it is unlikely that a small number of additional bases will resolve this. For reads between these extremes, the marginal benefit of a small number of additional bases is considerable, as these extra bases may make the difference between an ambiguous and an informative read.

A logistic curve was chosen to implement this scoring behavior, as it gives a relatively flat score for extreme values, while providing a steep transition around the user-specified threshold point. Given a target length *t*, the putative trimming to length *l* would give a length threshold score:

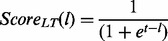



The second factor models ‘coverage’, and provides a linear score based on retained sequence length:

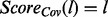



This reflects that, given reasonably high-accuracy bases, a longer read contains more information that is useful for most applications.

The final factor models the ‘error rate’, and uses the error probabilities from the read quality scores to determine the accumulated likelihood of errors over the read. To calculate this score, we simply take the product of the probabilities that each base is correct, giving:

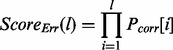



The correctness probabilities *P*_corr_ of each base are calculated from the sequence quality scores. The error score typically begins as a high score at the start of the read, and depending on the read quality, typically drops rapidly at some point during the read.

The Maximum Information algorithm determines the combined score of the three factors for each possible trimming position, and the best combined score determines how much of the read to trim. A user-specified strictness setting *s*, which can be set between 0 and 1, controls the balance between the ‘coverage’ factor (maximal for s = 0) and the ‘error rate’ factor (maximal for s = 1). This gives the following overall formula:

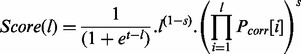



**Fig. 3.**
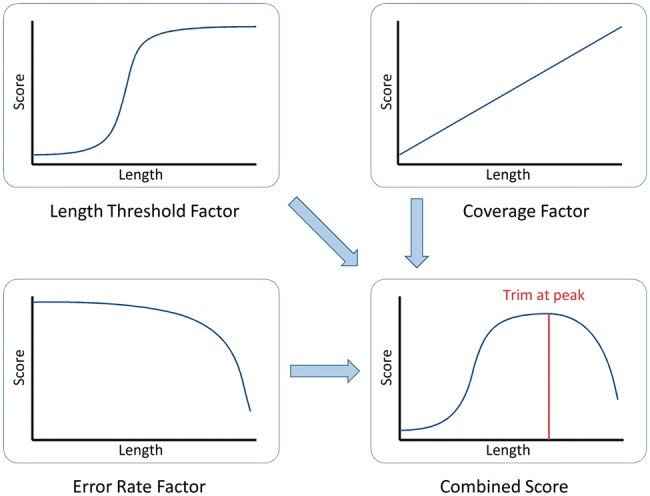
How Maximum Information mode combines uniqueness, coverage and error rate to determine the optimal trimming point

Figure 3 illustrates how the three factors are combined into a single score. The peak score is then used to determine the point where the read is trimmed.

## 3 IMPLEMENTATION

Trimmomatic uses a pipeline-based architecture, allowing individual ‘steps’ (adapter removal, quality filtering, etc.) to be applied to each read/read pair, in the order specified by the user. Each step can choose to work on the reads in isolation, or work on the combined pair, as appropriate. The tool tracks read pairing and stores ‘paired’ and ‘single’ reads separately.

A full list of the additional trimming and filtering steps is given in the Supplementary Materials and the online manual.

### 3.1 Convenience features

Input and output files can be specified individually on the command line, but for paired-end mode, where two similarly named input and four similarly named output files are often used, a ‘template’ name can be given instead of the input and/or output files. This template is automatically expanded to give the complete set of files needed. See Supplementary Materials for more details.

Compressed input and output are supported using either gzip or bzip2 formats. Compression/decompression is applied automatically when the appropriate file extensions are used, e.g. gz or bz2.

Performance can be improved using multiple threads if multiple CPU cores are available. The number of threads to use can be specified by the user or will be determined automatically if unspecified.

Trimmomatic supports sequence quality data in both standard (phred+33) and Illumina ‘legacy’ formats (phred+64), and can also convert between these formats if required. The quality format is determined automatically if not specified by the user.

The trimming status of each read can optionally be written to a log file. This is intended to help tune the choice of processing parameters used, but because it has a significant performance impact, it is not recommended unless needed.

## 4 RESULTS

To illustrate the value of data preprocessing, we evaluated two different scenarios: reference-based alignment using Bowtie 2 (Langmead and Salzberg, 2012) and BWA (Li and Durbin, 2009) against the *Escherichia coli* K-12/MG1655 reference (NCBI sequence NC_000913.2), and *de novo* assembly using Velvet (Zerbino and Birney, 2008), on public *E.coli* K-12/MG1655 datasets (SRA datasets SRX131047 and SRR519926), as described in the Supplementary Methods.

### 4.1 Reference-based alignment

Dataset 1 (SRX131047) represents a typical Illumina library, sequenced on the HiSeq 2000 using 2 × 100 bp reads. Quality checking with FastQC revealed a notable quality drop in many reads after cycle 75 in both but did not report a high level of adapter contamination.

In the reference-based scenario, preprocessing increased the number of uniquely aligned reads from dataset 1, as seen in the first portion of Table 1. Filtering for both adapters and quality achieves the best result, and quality trimming is especially important when alignment settings are strict. The Maximum Information approach outperforms the Sliding Window approach in both cases, with a wider margin when the alignment mode is strict.

**Table 1.** Results of alignment of raw data and data trimmed by Trimmomatic from both datasets

Dataset/aligner	Reads	Tolerant^a^	Strict^b^
Dataset 1 with Bowtie2 aligner			
Unfiltered	11 008 190	9 018 810	6 401 927
Trimmomatic—adapters only	11 008 150	9 117 952	6 510 253
Trimmomatic—SW	9 456 826	9 079 434	8 086 905
Trimmomatic—MI	9 456 826	9 116 627	8 748 376
Trimmomatic—adapters and SW	9 456 819	9 150 361	8 111 470
Trimmomatic—adapters and MI	9 456 126	**9 153 375**	**8 748 401**
Dataset 1 with BWA aligner			
Unfiltered	11 008 190	8 750 851	7 834 544
Trimmomatic—adapters only	11 008 150	8 864 884	7 942 198
Trimmomatic—adapters and SW	9 456 819	9 110 831	8 810 063
Trimmomatic—adapters and MI	9 456 126	**9 145 423**	**9 056 403**
Dataset 2 with BWA aligner			
Unfiltered	801 192	60 010	11 592
Trimmomatic—adapters only	801 164	121 926	68 177
Trimmomatic—adapters and SW	655 075	628 867	590 729
Trimmomatic—adapters and MI	658 796	**639 740**	**634 779**

*Note*: Adapter trimming, where done, used palindrome mode. Best values per dataset and aligner are indicated in bold. MI indicates Maximum Information mode, and SW indicates Sliding Window mode.

^a^Alignment allowing some mismatches and/or INDELs. See Supplementary Methods for more details.

^b^Aligned when no mismatches or INDELs were allowed.

Notably, the optimal results for strict alignment and tolerant alignment were found using widely different quality stringency settings. (See Supplementary Results for more details.)

To validate these results with an alternative aligner, we repeated the experiment using BWA. Although the alignment counts differ, because of slight differences between the tools in the settings or algorithms, the overall trend is similar. The best results are again achieved when filtering for both adapters and quality, as shown in the second part of Table 1.

Dataset 2 (SRR519926) is a 2 × 250 bp run, sequenced on an MiSeq. Although read quality is high at the start of each forward read, the longer read length allows more opportunity for errors to accumulate in the lower quality final 60–70 bases of each read. Furthermore, the reverse read has notably poorer quality, with quality dropping significantly by approximately base 120. These quality issues can be seen clearly in the FastQC plots, shown in the Supplementary Figure S1, compared with the much higher average quality of the post-filtered data, as shown in Supplementary Figure S2.

Not surprisingly, trimming is even more critical to achieving acceptable alignment rates with these data. The final part of Table 1 shows that <1.5% of the reads align in strict mode, which requires a perfect match, while just 7% of the reads can be aligned when allowing for one mismatch. Even with the liberal default settings, allowing nine mismatches, <25% (197 933 reads) can be aligned. However, after trimming, almost 78% of the reads align perfectly.

### 4.2 *De novo* assembly

Both datasets also showed considerable improvement in a *de novo* assembly scenario. For the first dataset, the contig N50 size increased by 58% (95 389 versus 60 370 bp) after preprocessing, while the maximum contig size improved by ∼28%. Also, the assembly from unfiltered data contained a 34-bp perfect match to an adapter sequence, while no adapters were found in the filtered assemblies.

The second dataset showed even greater benefits after trimming, with ∼77% improvement in N50 contig size (177 880 versus 100 662 bp) and ∼55% increase in maximum contig size. Perhaps surprisingly, no adapter sequences were found in the assembly of the untrimmed version of this dataset.

### 4.3 Comparison with existing tools

We also compared the performance of Trimmomatic with a variety of existing adapter and quality filtering tools in similar referenced-based scenarios, as described in the Supplementary Methods. The tools selected were AdapterRemoval (Lindgreen, 2012), and Scythe/Sickle (https://github.com/najoshi/), which fully support paired-end data and EA-Utils (Aronesty, 2013), which maintains read pairing but loses singletons (reads whose mate has been filtered). Additionally, the single-end tools Cutadapt (Martin, 2011), Fastx-Toolkit (http://hannonlab.cshl.edu/fastx_toolkit) and Reaper (http://www.ebi.ac.uk/∼stijn/reaper) were included.

We filtered and aligned using paired-end mode for those tools that support it, but we used single-end mode as a fallback where necessary. In practice, ignoring pairing will result in suboptimal alignments but was done here in the interest of making the output of all tools comparable.

Table 2 shows the output of the various tools aligned using Bowtie 2 in both tolerant and strict alignment settings. The top portion of this table, which shows the results using a tolerant alignment, suggests that the best tools perform almost identically in terms of output quality, with <20 000 reads separating the top three, and most tools within ∼1% of the best. However, given that the unfiltered data show a difference of just 1.5%, the narrowness of the result is likely due to the relatively low rate of adapter contamination in this dataset, the high average read quality and the tolerant alignment settings used.

**Table 2.** Results of Bowtie2 alignment of dataset 1 showing raw data and the trimmed data by each tool

Dataset/alignment	Reads	Alignment (paired)^a^	Run time^b^ (s)
Tolerant alignment			
Unfiltered	11 008 190	9 018 810 (8,323,786)	N/A
Fastx-Toolkit	9 631 977	8 073 757 (N/A)	670.1/356.3
Reaper	9 428 331	9 057 448 (N/A)	324.8/166.8
Cutadapt	9 456 172	9 127 667 (N/A)	342.5/176.7
EA-Utils	8 995 134	8 662 596 (8 578 790)	9.3/**8.0**
Scythe/Sickle	9 453 459	9 133 464 (8 636 984)	529.3/279.7
AdapterRemoval	9 456 350	9 147 915 (8 689 668)	960.2
Trimmomatic SW	9 456 819	9 150 361 (8 693 000)	33.7/9.6
Trimmomatic MI	9 456 819	**9 153 375** (**8 697 690**)	34.3/9.7
Strict alignment			
Unfiltered	11 008 190	6 401 927 (4 857 606)	N/A
Fastx-Toolkit	8 263 345	7 187 257 (N/A)	—
Reaper	9 355 765	8 010 326 (N/A)	—
Cutadapt	9 390 371	8 086 428 (N/A)	—
EA-Utils	8 910 356	7 757 108 (7 056 242)	—
Scythe/Sickle	9 339 668	8 060 612 (6 993 076)	—
AdapterRemoval	9 454 189	8 103 596 (7 050 788)	—
Trimmomatic SW	9 355 985	8 111 470 (7 068 406)	—
Trimmomatic MI	9 456 124	**8 748 401** (**8 053 230**)	—

*Note*: Both quality modes are shown for Trimmomatic. Best values are indicated in bold. MI indicates Maximum Information mode, and SW indicates Sliding Window mode.

^a^Total reads aligned, and the subset that are aligned as pairs.

^b^Shows wall time, for both serial and parallel execution. See Supplementary Methods for more details.

The execution time varies widely, with EA-Utils leading, Trimmomatic following closely, while the remaining tools require considerably longer time. However, the testing methodology, using the median of 3 runs on a relatively small dataset, allows the entire dataset to be cached. In practice, it is likely that at least the faster tools will be limited by IO performance. The individual execution times for each run are shown in Supplementary Table S4.

The equivalent alignment using strict mode, shown in the bottom part of the table, more clearly differentiates the tools, with the margin between Trimmomatic in Maximum Information mode and the alternatives widening considerably.

Alignments of the same dataset using BWA painted a broadly similar picture, as shown in the top half of Table 3, although the difference between strict and tolerant mode is not so strong. Again, Maximum Information mode appears to outperform by a greater margin for stricter alignments.

**Table 3.** Results of strict and tolerant BWA alignments of the raw data and trimmed data from each tool (using both quality modes for Trimmomatic) from both datasets

Dataset	Strict alignments^a^	Tolerant alignments^b^
Dataset 1		
Unfiltered	7 834 544	8 750 851
Fastx-Toolkit	7 187 257	7 894 580
Reaper	8 010 326	8 894 757
Cutadapt	8 086 428	8 968 519
EA-Utils	8 059 850	8 896 724
Scythe/Sickle	8 755 676	9 076 936
AdapterRemoval	8 810 051	9 108 691
Trimmomatic SW	8 810 063	9 110 831
Trimmomatic MI	**9 056 403**	**9 145 423**
Dataset 2		
Unfiltered	11 592	60 010
AdapterRemoval	513 133	574 973
Fastx-Toolkit	525 519	550 695
EA-Utils	538 472	588 046
Scythe/Sickle	567 976	588 135
Cutadapt	568 044	613 089
Trimmomatic SW	590 729	628 867
Trimmomatic MI	**634 779**	**639 740**

*Note*: Best values are indicated in bold. MI indicates Maximum Information mode, and SW indicates Sliding Window mode.

^a^Reads aligned, zero mismatches permitted.

^b^Reads aligned, one mismatch allowed.

The results of dataset 2, shown in the lower half of Table 3, rank many of the tools differently, with AdapterRemoval dropping significantly in the ranking. Trimmomatic remains the best performer, especially in Maximum Information mode, but Cutadapt becomes the closest challenger. Reaper was unable to process this dataset, perhaps because of the long read length.

## 5 DISCUSSION

### 5.1 The need for read preprocessing

We have illustrated the advantages of NGS data preprocessing in both reference-based and *de novo* assembly applications. For high-quality datasets, in reference-based applications, the benefits of preprocessing seem somewhat limited. We show ∼1.5% gain in unique alignments shown if mismatch tolerant aligner settings are used, although a more substantial difference could be seen when perfect matches were required. In practice, however, given a high-quality dataset like this, the benefits to a downstream application such as variant calling are likely to be small.

The second dataset, which had reads with substantially lower quality, illustrated that even reference-based tasks can benefit substantially from read preprocessing. Less than 25% of reads could be aligned by BWA without preprocessing. This could be improved to almost 80% by preprocessing, with almost 78% aligning even with strict settings.

In the *de novo* assembly scenario, trimming was needed to ensure adapter sequences would not be incorporated into the newly assembled genome. This benefit was accompanied by a significant 58%/77% improvement in N50, respectively, and 28%/55% improvement, respectively, in maximum contig size for two datasets. The substantial improvement in assembly statistics further justifies the preprocessing of reads for *de novo* assembly.

It is perhaps not surprising that preprocessing is so beneficial to *de novo* assembly, as many assembly tools, including velvet, do not exploit quality scores and thus treat all data equally, regardless of the known difference in quality. The effect of adapter sequences is also more serious, given the risk of incorporating adapter sequences into the final sequence assembly, compared with the mere reduction in the alignment rate typically seen in reference-based approaches.

### 5.2 Comparison against existing tools

Trimmomatic compared favorably against all other tools in the tests performed.

When using high-quality raw data and liberal alignment criteria, the differences between the tools were relatively small. In this scenario, AdapterRemoval performed particularly well, reflecting its relative strength in removing technical sequences. This is unsurprising because, to the authors’ knowledge, AdapterRemoval is the only other tool to implement a pair-aware adapter removal strategy.

Nonetheless, the use of strict alignment criteria, especially when combined with poor-quality input data, allows the differences between the tools to become clearer. In these scenarios, appropriate trimming based on quality seems to be more important, whereas technical sequence identification appeared to matter less. This helps explain the change in relative rankings of the tools between the two datasets. Trimmomatic with the Maximum Information mode seems to perform exceptionally well in these challenging scenarios.

*Funding*: We want to thank the BMBF for funding through grants 0315702F, 0315961 and 0315049A and BLE/BMELV Verbundprojekt: G 127/10 IF.

*Conflict of Interest*: none declared.

## Supplementary Material

Supplementary Data
