# Supplementary material for: Trimmomatic: a flexible trimmer for Illumina sequence data
Source: Bioinformatics. 2014 Apr 1;30(15):2114–20. doi: 10.1093/bioinformatics/btu170 (PMC4103590; doi:10.1093/bioinformatics/btu170)
Supplement: Supplementary Data [file supp_30_15_2114__index.html]

Trimmomatic: A flexible trimmer for Illumina Sequence Data — Trimmomatic: a flexible trimmer for Illumina sequence data — Trimmomatic: a flexible trimmer for Illumina sequence data — Trimmomatic: a flexible trimmer for Illumina sequence data — Supplementary Data 

# Trimmomatic: a flexible trimmer for Illumina sequence data

## Supplementary Data

files

**Files in this Data Supplement:**

- Supplementary Data - txt file
- Supplementary Data - docx file
