# Supplementary material for: Trimmomatic: a flexible trimmer for Illumina sequence data
Source: Bioinformatics. 2014 Apr 1;30(15):2114–20. doi: 10.1093/bioinformatics/btu170 (PMC4103590; doi:10.1093/bioinformatics/btu170)
Supplement: Supplementary Data [file supp_btu170_Trimmomatic-Sup.docx]

# SUPPLEMENTARY IMPLEMENTATION

To cover unique user requirements, Trimmomatic includes the following additional processing steps, which are algorithmically quite simple. However, by integrating them into the Trimmomatic pipeline, they can be applied as required, while maintaining the pair-awareness and other advantages of integration into a single tool.

## Quality Filtering

In addition to the sliding window and maximum information trimmers already discussed, Trimmomatic includes simple 5’ and 3’ end-trimmers. These start at the specified end of the read, and remove a successive series of bases which are all below the specified quality score. These trimmers are primarily designed for removal of artifacts, such as ‘N’ base calls or a series of ‘B’ quality scores which were introduced by the 1.3 Illumina pipeline. These modes are not intended for quality filtering, but rather for removing artifacts which may cause sub-optimal trimming by the sliding window or maximum information trimming steps.

## Read Cropping

Cropping removes bases from reads, regardless of quality or content. The ‘CROP’ step cuts all reads to the specified length, while the ‘HEADCROP’ step removes the specified number of bases from the start of each read.

## Read Filtering

Read filtering drops a read entirely if it does not meet the specified criteria. Currently supported criteria are ‘MINLEN’, which requires that a read be of a specified minimal length, and ‘AVGQUAL’ which requires that the read have a specified average quality across all bases.

## Quality Score Conversion

Quality score conversion simplifies pipelines which have to deal with a combination of Phred+33 and Phred+64 data, allowing downstream tools to treat data uniformly. Conversion is supported in both directions.

# Supplementary Methods

## Reference-based Test

The reference based test was designed to illustrate the value of pre-processing NGS reads before aligning them to a known reference sequence. Typical examples of this kind of workflow include genome variant discovery and RNA-Seq.

The chosen metric for this scenario was the number of validly aligned reads and/or pairs. It is reasonable to infer that a greater number of aligned reads would translate to greater sensitivity for detecting genomic variation, or in the case of RNA-Seq data, differential expression.

The aligner and alignment settings chosen have a dramatic influence on how much pre-processing is optimal. For extremely liberal alignment settings, which are the default for Bowtie2 (version 2.1.0), adapter trimming alone without any quality filtering was optimal (data not shown). For moderately tolerant settings (--score-min L,-0.1,-0.1), most trimming tools resulted in improvements, as shown in the top half of Table 2 in the main text. The most dramatic benefits of trimming were found for strict alignments, allowing no errors (--score-min L,0,0), as shown in bottom half of Table 2 in the main text.

For BWA, alignments were done allowing one error in tolerant mode (-n 1), and zero errors in strict mode (-n 0). The default settings, allowing up to 9 errors in a 250bp read, were also used to confirm the poor results of the second dataset if no preprocessing was done.

## Comparison of Single-end and Paired-end processing

In the main text, we argue that paired end data offers theoretical advantages for adapter removal, as it can provide a better sensitivity/specificity trade-off. It is also commonly considered to be of benefit during alignment, where the potential alignments of each read in the pair can help constrain possible placements of the other read, and thus the combination can be more accurately placed than the reads in isolation. Nonetheless, it is important to verify that these theoretical advantages translate into practice in real data.

This test illustrates the advantages offered by processing the data in a ‘pair-aware’ manner, for both adapter removal and alignment. The data was either untrimmed, trimmed by Trimmomatic using ‘palindrome’ mode which takes advantage of read pairing, or trimmed using ‘simple’ mode which ignores pairing. In each case, the data was also quality-trimmed using the Sliding Window approach, using the optimal settings previously determined above. The resulting data were aligned using bowtie2 using strict settings (no mismatches or INDELs), using both paired-end and single-end modes.

## *De Novo* Assembly Test

The *de novo* assembly test was designed to confirm the value of pre-processing for tasks without a reference sequence. A *de novo* assembly is particularly sensitive to adapter and other technical sequences within the reads since they are liable to include such sequences in the output, e.g. a genome assembly.

Even random base-call errors serve to complicate the assembly process, and typically increase the memory and processing time required to assemble the genome considerably. In most cases, the assembler is not able to fully correct or remove these errors, and thus the final assembly quality is reduced.

Assessing the quality of a *de novo* assembly is difficult, since typical metrics give only limited insight and cannot determine correctness, especially since a known reference is usually not available. However metrics such as N50, maximum contig length or contig number can be used to access the degree of assembly fragmentation, while total contig or scaffold size can be used to indicate how complete the assembly is, relative to the known (or estimated) genome size.

Assemblies were created using Velvet 1.2.08, using a K-mer size of 21. Expected coverage was calculated by the dataset size, using a rough 5 megabase genome size estimation, to more realistically reflect real world *de novo* assembly scenarios, since the exact genome size would usually not be available. The minimum coverage cut-off was set to 20% of the expected coverage in each case.

## Comparison to Existing Tools

The reference-based scenario was repeated using a selection of existing pre-processing tools. Since some of these tools are not pair-aware and thus do not maintain pair correspondence, we evaluated the filtered data using single-ended mode as a fall-back where necessary.

For each tool, we aligned the dataset 1 using bowtie2, with both ‘strict’ and ‘tolerant’ settings as described above. To confirm the general applicability of the results to other aligners and datasets, we repeated the alignment of dataset 1 using BWA, and also aligned a second dataset, with BWA only.

The default or recommended adapter detection settings were used for each tool, and the output filtered to remove reads shorter than 36bp. A wide range of quality stringency settings were tested for each tool to find the maximum number of uniquely aligning reads.

Runtime was measured in ‘serial’ mode (one process without threading) and ‘parallel’ mode (up to 8 processes/threads where possible). For tools which processed the forward and reverse reads in isolation, but lacked internal support for parallelization, parallel mode was achieved by running forward and reverse read processing simultaneously. All timings are the median of 3 consecutive runs, and were carried out on an otherwise idle Intel Xeon E3-1270V2, running at 3.5GHz, using 64-bit Linux. No substantial variability between the runs was found.

# supplementary RESULTS

## Reference-based Test

The optimal settings for quality filtering were found to be strongly dependent on the error-tolerance settings of the downstream aligner.

With Bowtie2, and using the Sliding Window trimmer, quality 13 was optimal for ‘strict’ alignment, while even the most lenient setting of Sliding Window was found to be counter-productive for ‘tolerant’ alignment. The Maximum Information trimmer was optimal using a relatively liberal 0.4 stringency setting for ‘tolerant’ alignments, while a very high 0.999 stringency setting was found to be optimal for ‘strict’ alignment.

**Supplementary Table 1.**Results of ‘strict’ alignment of the data filtered using a range of settings around the optimum setting, for both Sliding Window (SW) and Maximum Information (MI) mode. The best values are in bold.

| Filtering | Reads | Aligned (Paired)^(1)^ | Ambiguous | Unaligned |
| --- | --- | --- | --- | --- |
| SW Q10 | 9,438,021 | **8,102,192 (7,047,300)** | **134,512** | **1,201,317** |
| SW Q11 | 9,425,289 | **8,105,721 (7,052,980)** | **134,595** | **1,184,973** |
| SW Q12 | 9,400,281 | **8,109,946 (7,060,966)** | **134,622** | **1,155,613** |
| SW Q13 | 9,355,985 | **8,111,470 (7,068,406)** | **134,773** | **1,109,742** |
| SW Q14 | 9,287,107 | **8,104,896 (7,070,054)** | **134,798** | **1,047,413** |
| SW Q15 | 9,192,615 | **8,084,241 (7,059,260)** | **134,621** | **973,753** |
| MI 0.995 | 9,456,125 | **8,746,356 (8,048,678)** | **161,641** | **548,128** |
| MI 0.996 | 9,456,124 | **8,747,486 (8,051,248)** | **162,170** | **546,468** |
| MI 0.997 | 9,456,124 | **8,748,100 (8,052,606)** | **162,444** | **545,580** |
| MI 0.998 | 9,456,124 | **8,748,147 (8,052,692)** | **162,455** | **545,522** |
| MI 0.999 | 9,456,124 | **8,748,401 (8,053,230)** | **162,554** | **545,169** |

^(1)^ Total reads uniquely aligned, and the subset which are aligned as pairs.

Finding the optimal trimming level for reference-based tasks proved relatively straightforward, since there is typically a single local optimum which is also the global optimum. Furthermore, relatively small differences can be seen between the optimum and nearby values, so using a value near the optimum is not likely to dramatically change the results of downstream analysis.

## Comparison of Paired End and Single End processing

These results clearly illustrate that, in practice, paired-end adapter trimming offers considerable advantages over single-end adapter trimming, while paired-end alignment provides additional gains, albeit more moderate in this particular dataset.

**Supplementary Table 2.**Results of paired-end vs. single-end processing for both adapter trimming and alignment. The best values are in bold.

| Adapter Trimming | Reads | Alignment | Aligned (Paired)^(1)^ |
| --- | --- | --- | --- |
| Unfiltered | 11008190 | Single-End | **6389691 (N/A)** |
|  |  | Paired-End | **6401927 (4857606)** |
| Single-End | 9355863 | Single-End | **8015443 (N/A)** |
|  |  | Paired-End | **8033953 (6975626)** |
| Paired-End | 9355985 | Single-End | **8093095 (N/A)** |
|  |  | Paired-End | **8111470 (7068406)** |

^(1)^ Total reads aligned, and the subset which are aligned as pairs.

## *De Novo* Assembly Test

*De novo* assemblies also show dramatic improvements from both adapter and quality filtering. However, unlike reference-based tasks, there is no single natural metric for *de novo* assembly, with N50, maximum sequence length and total sequence length each providing different information. Checking for contaminants, such as adapter sequences, is also important.

**Supplementary Table 3.***De novo* assembly results of the raw data and various levels of quality filtering. The best values are in bold for

| Dataset | N50 | Maximum Length | Total length | Adapters in Assembly |
| --- | --- | --- | --- | --- |
| Unfiltered | 60,370 | 278,485 | 4,559,130 | Y |
| Adapters only | 60,570 | 356,432 | 4,558,257 | **N** |
| Leading/Trailing | 80,442 | 356,348 | 4,557,936 | **N** |
| SW Q10 | 80,385 | **356,374** | 4,557,504 | **N** |
| SW Q11 | 85,771 | **356,384** | 4,556,446 | **N** |
| SW Q12 | 87,413 | **356,261** | 4,557,728 | **N** |
| SW Q13 | 80,366 | **356,380** | 4,557,801 | **N** |
| SW Q14 | 82,701 | **356,391** | 4,559,711 | **N** |
| SW Q15 | 87,346 | **356,246** | 4,557,153 | **N** |
| MI 0.990 | 94,655 | **356,542** | 4,559,237 | **N** |
| MI 0.992 | 95,320 | **356,415** | 4,558,774 | **N** |
| MI 0.994 | **95,389** | **356,451** | 4,557,981 | **N** |
| MI 0.996 | 94,012 | **356,576** | 4,557,506 | **N** |
| MI 0.998 | 95,339 | **356,472** | 4,557,006 | **N** |


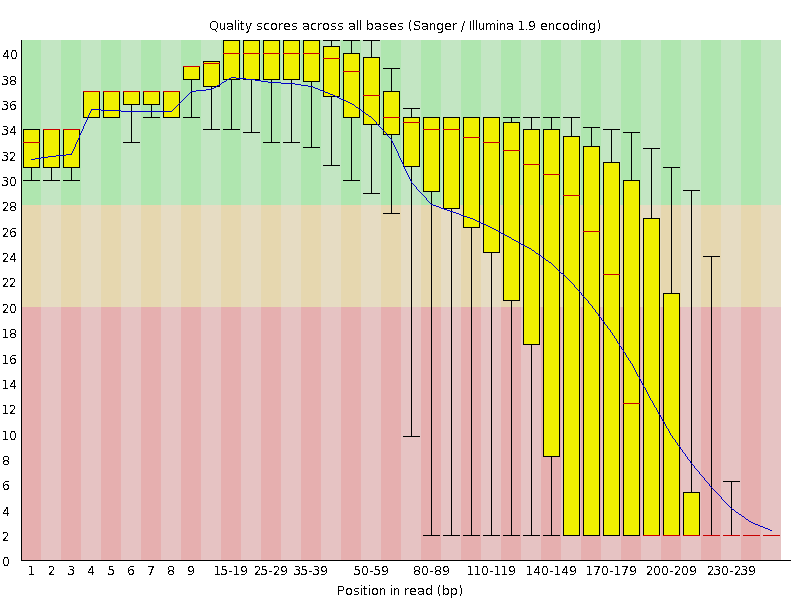
Interestingly, of the three metrics, only the N50 seems to dramatically vary with differing quality filtering levels. Both Sliding Window and Maximum Information approaches substantially improve the N50 compared to the unfiltered datasets, with the latter offering the biggest improvement.

Unlike the reference-based alignment scenario, finding the optimal level of trimming for *de novo* assembly appears challenging. Regardless of the trimming method used, the N50 score is shown to be discontinuous around the global optimum, and additional local optima can be seen. Furthermore, the N50 values achieved using settings adjacent to the optimal setting vary considerably, especially when using the Sliding Window approach.


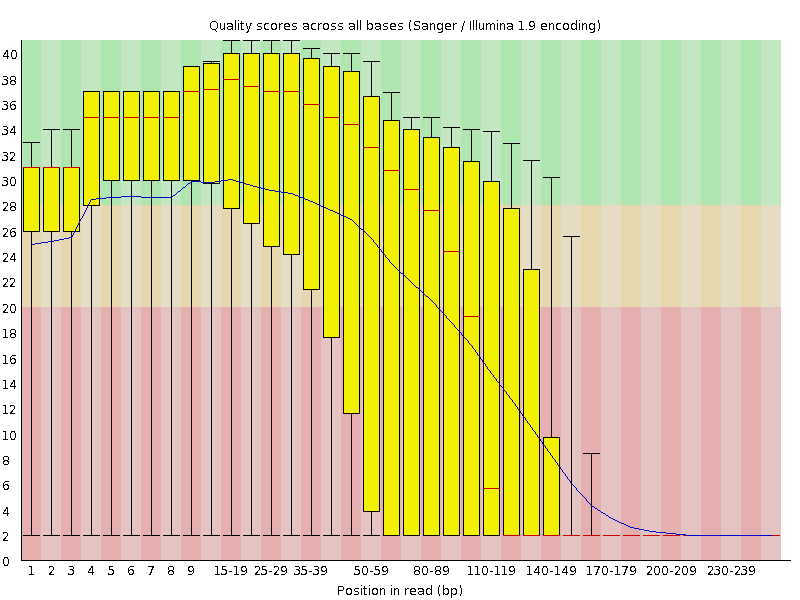


## Comparison to Existing Tools: Execution times

The full results of the execution time comparison are shown in Supplementary table 4.

**Supplementary Table 4.**Execution time of tools trimming dataset 1, using optimal parameters. Tools were tested using both serial and parallel mode where possible.

| Dataset | Serial/Parallel^(1)^ | Run 1 | Run 2 | Run 3 | | |
| --- | --- | --- | --- | --- | --- | --- |
| Fastx Tookit  Fastx Tookit | Serial | 670.1s | 668.0s | 678.6s | | |
|  | Parallel | | 353.7s | 356.3s | 359.4s |  |
| Reaper | | Serial | | 324.8s | 323.9s | 326.9s |
| Reaper | | Parallel | | 167.0s | 165.6s | 166.8s |
| Cutadapt | | Serial | | 339.4s | 344.4s | 342.5s |
| Cutadapt | | Parallel | | 176.7s | 177,1s | 175.9s |
| EA-Utils | | Serial | | 9.3s | 9.4s | 9.3s |
| EA-Utils | | Parallel | | 8.0s | 8.0s | 8.0s |
| Scythe/Sickle | | Serial | | 531.9s | 529.3s | 524.7s |
| Scythe/Sickle | | Parallel | | 278.5s | 280.0s | 279.7s |
| AdapterRemoval | | Serial | | 962.1s | 960.2s | 953.8s |
| Trimmomatic SW | | Serial | | 33.7s | 33.9s | 33.4s |
| Trimmomatic SW | | Parallel | | 9.7s | 9.6s | 9.6s |
| Trimmomatic MI | | Serial | | 34.6s | 34.3s | 34.1s |
| Trimmomatic MI | | Parallel | | 9.7s | 9.6s | 9.7s |

^(1)^ Serial mode successive execution (if needed) of serial processes. Parallel mode indicates parallel processes or multithreaded execution where possible.

## FastQC report of pre/post filtered data

The severity of quality issues in dataset 2 can be seen relatively easy using FastQC. Supplementary Figure 1 shows the average quality of across the read for the untrimmed data, while Supplementary Figure 2 shows the same data after Maximum Information trimming. Not surprisingly, the number of aligning reads is much greater post-filtering, as shown in the second part of Table 3 in the main text.**
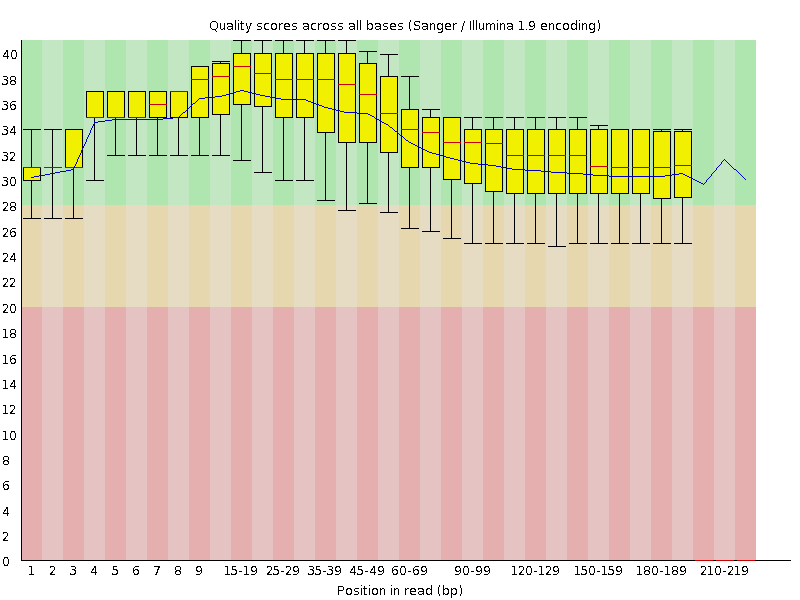
**

**Supplementary Fig. 1.**FastQC plot of quality score vs position in the untrimmed data.
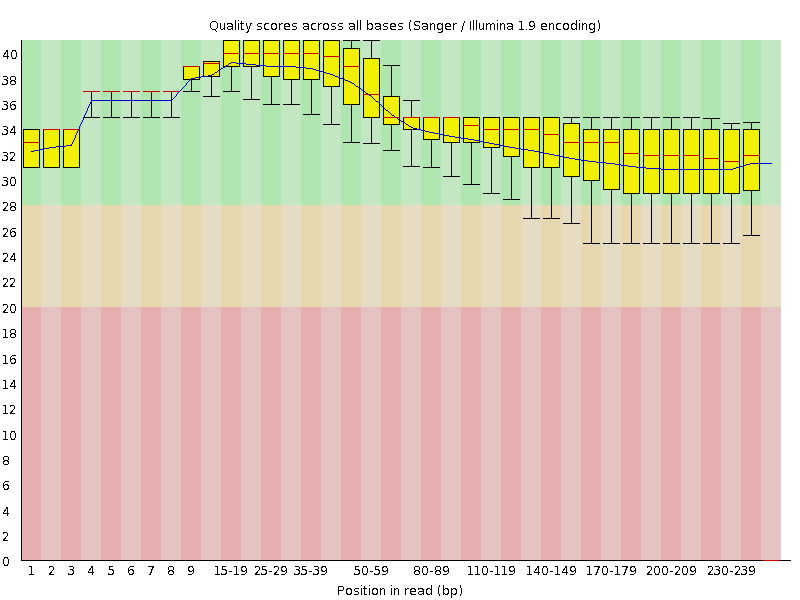


**Supplementary Fig. 2.**FastQC plot of quality score vs position in the data trimmed using the optimal settings of Maximum Information mode.
